# Anaesthetic Management and Multidisciplinary Approach in a Case of Aortic Foreign Body Impalement Following Thoracolumbar Instrumentation

**DOI:** 10.4274/TJAR.2025.252014

**Published:** 2026-02-09

**Authors:** Burhan Dost, Esra Turunç, Belkıs Eroğlu Çelik, Yunus Emre Durmuş, Mustafa Kemal Demirağ

**Affiliations:** 1 Ondokuz Mayıs University Faculty of Medicine, Department of Anaesthesiology and Reanimation, Samsun, Türkiye; 2 Ondokuz Mayıs University Faculty of Medicine, Department of Neurosurgery, Samsun, Türkiye; 3 Ondokuz Mayıs University Faculty of Medicine, Department of Cardiovascular Surgery, Samsun, Türkiye

**Keywords:** Cardiovascular and thoracic anaesthesia, neuroanaesthesia, spinal instrumentation, TEVAR, thoracic aortic injury

## Abstract

Iatrogenic thoracic aortic injury caused by misplaced spinal instrumentation is a rare but potentially fatal complication of posterior spinal fusion and fixation procedures. The close anatomical relationship between the vertebral column and descending thoracic aorta puts the aortic wall at risk, especially when pedicle screws are malpositioned. While such injuries may remain asymptomatic initially, progressive erosion of the aortic wall can lead to catastrophic rupture. This case report highlights a 72-year-old woman with a history of diabetes, hypertension, and Takotsubo cardiomyopathy who developed a thoracic aortic injury following thoracolumbar instrumentation. Imaging revealed a pedicle screw at the T5 level, directly impinging on the aortic wall. A multidisciplinary approach involving cardiovascular, neurosurgery, and anaesthesiology teams was utilized, and thoracic endovascular aortic repair (TEVAR) was performed to stabilize the aorta before hardware removal. Despite successful surgical intervention, the patient later developed a right-sided middle cerebral artery infarction, possibly due to thromboembolism from the TEVAR site. This case underscores the importance of a staged surgical approach with TEVAR in managing aortic injury during spinal instrumentation, especially in high-risk patients with comorbidities such as Takotsubo cardiomyopathy. Careful anaesthesia management and multidisciplinary collaboration are essential to optimize outcomes in such complex cases.

**Main Points**

• Iatrogenic thoracic aortic injury from misplaced spinal instrumentation requires urgent intervention.

• Thoracic endovascular aortic repair is effective for stabilizing the aorta before hardware removal in high-risk patients.

• A multidisciplinary approach is essential for managing complex cases with multiple comorbidities.

• Postoperative neurological risks, like thromboembolic events, must be carefully monitored.

## Introduction

Thoracic aortic injuries caused by misplaced spinal instrumentation are rare but potentially fatal complications of posterior spinal fusion and fixation procedures. The close anatomical relationship between the vertebral column and descending thoracic aorta places the aortic wall at risk during instrumentation, particularly when posterior pedicle screws are malpositioned. Although such injuries may remain clinically silent for a period of time, progressive erosion of the aortic wall by hardware can ultimately result in life-threatening rupture. The diagnosis and management of these injuries are further complicated by variability in clinical presentation, ranging from incidental radiological findings to acute hemorrhage. In recent years, thoracic endovascular aortic repair (TEVAR) has emerged as a less invasive and effective strategy for stabilizing the aorta before hardware removal, thereby reducing the risk of catastrophic bleeding.^1^

Here, we present a case of iatrogenic aortic impingement caused by a pedicle screw, emphasizing  anaesthetic challenges and perioperative decision-making  in the context of multiple comorbidities, including Takotsubo cardiomyopathy  with severely reduced myocardial function.

## Case Report

**Figure 1:**
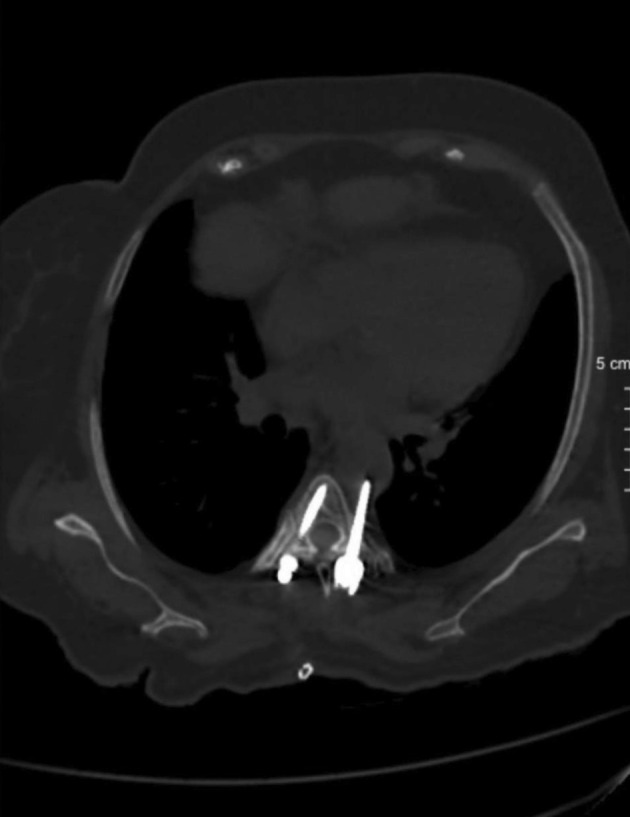
Axial computed tomography scan of the thorax demonstrating a malpositioned left-sided pedicle screw at the T5 level in close proximity to the descending thoracic aorta. The screw is seen penetrating the left lateral aspect of the vertebral body, creating an indentation on the adjacent aortic wall, indicating direct contact and risk of vascular injury.

A 72-year-old female with a history of diabetes mellitus and hypertension was immobilized for 1.5 years due to paraplegia, which developed postoperatively following scoliosis correction and lumbar decompression surgery. She subsequently underwent thoracolumbar instrumentation after which she was admitted to the intensive care unit with ST-segment elevations and elevated troponin levels, raising the suspicion of inferolateral myocardial infarction. Coronary angiography revealed normal coronary arteries, while echocardiography demonstrated global hypokinesis with a left ventricular ejection fraction of 30-35%. It also showed apical segment hypokinesis and hypercontractility of the basal segments, leading to a diagnosis of Takotsubo cardiomyopathy. Routine postoperative imaging, including thoracic computed tomography, revealed a pedicle screw at the T5 level, penetrating the left lateral aspect of the vertebral body, with direct contact and indentation of the adjacent aortic wall (Figure 1). Given the high-risk of aortic rupture, a multidisciplinary approach involving cardiovascular surgery, neurosurgery, and anaesthesiology teams is required. Despite the absence of hematoma, pseudoaneurysm formation, or pleural effusion, it was evident that the misplaced pedicle screw resulted in aortic injury. Nevertheless, based on radiological findings alone, it was challenging to determine whether the screw tip had breached the posterior thoracic wall and entered the lumen or merely compressed the aortic wall. The team agreed to proceed with TEVAR to strengthen the affected section of the aorta.

Under general anaesthesia, radial artery catheterization and right internal jugular central venous catheter placement were performed. Induction and tracheal intubation were conducted cautiously to avoid hemodynamic instability, which could increase shear stress on the aortic wall. Anaesthesia was induced using propofol (0.5-2 mg kg^-1^), remifentanil (0.5 µg kg^-1^ IV bolus and 0.3 µg kg^-1^ min^-1^ infusion), and rocuronium (1 mg kg^-1 ^IV) for endotracheal intubation. Neuromuscular blockade was maintained intraoperatively with additional rocuronium boluses, targeting a Train-of-Four count of 1-2. Anaesthetic maintenance included sevoflurane in an oxygen and air mixture (60% O₂) at age-adjusted 1.0 minimum alveolar concentration, along with a continuous remifentanil infusion, both titrated to maintain mean arterial pressure (MAP) and heart rate within 20% of baseline values. The depth of anaesthesia was guided using bispectral index monitoring, with values maintained between 40 and 60. Volume-controlled ventilation was implemented with a tidal volume of 7-8 mL kg^-1^, an I:E ratio of 1:2, and respiratory rate adjustments to achieve an end-tidal CO₂ of 30-35 mmHg. Central venous access via the right internal jugular vein enabled additional hemodynamic monitoring and guided fluid management. Glycemic levels and fluid therapy were also carefully titrated according to intraoperative needs. Intraoperative hemodynamics remained stable, with no episodes of hypotension or arrhythmia observed, and no need for vasopressor or inotropic agents. In the preoperative period, thromboembolism prophylaxis was initiated with subcutaneous low molecular weight heparin and compression stockings, considering the patient’s prolonged immobility and elevated risk profile. A massive transfusion protocol was prepared in anticipation of potential catastrophic hemorrhage after screw removal. The surgical plan was staged to optimize patient safety and minimize the risk of aortic rupture. Immediately after anaesthesia induction and intubation, the cardiovascular surgery team deployed a TEVAR sheath through the common femoral artery to provide immediate endovascular control in cases of rupture during screw extraction. The patient was then turned to the prone position, allowing the neurosurgical team to safely extract the screw without applying excessive traction force that could destabilize the aortic wall. The screw was successfully removed, without bleeding. The patient was then carefully repositioned supine to facilitate the placement of a straight thoracic endovascular covered Ankura TAA stent graft (Lifetech Scientific, Shenzhen, China), reinforcing the structurally compromised aortic segment and preventing a delayed rupture. The patient was administered 5000 IU of low molecular weight heparin intravenously. The reason the TEVAR stent graft was not deployed before turning prone is related to the necessity to safely extract the pedicle screw and minimize the risk of destabilizing the aorta. It was crucial to ensure that the screw extraction process did not cause aortic rupture before the stent graft could be placed. This is why we did not deploy the graft beforehand. Throughout all stages of the procedure, the patient’s hemodynamic profile remained stable without the need for vasopressor or inotropic support. MAP was maintained between 65 and 80 mmHg, and heart rate remained within 70-85 beats per minute. Intraoperative fluid therapy included 1500 mL of balanced crystalloid solution, with no significant blood loss or need for transfusion.

The immediate postoperative course was uneventful, and the patient was extubated in the intensive care unit with stable hemodynamics. However, on postoperative day 2, she developed right-sided hemiparesis, and brain magnetic resonance imaging revealed an acute middle cerebral artery infarction. No atrial fibrillation was detected on continuous electrocardiography (ECG) monitoring, and echocardiography revealed no cardiac embolic sources. Given the timing and vascular distribution, we hypothesized that the infarct resulted from thromboembolism originating from the TEVAR deployment site. This highlights the under-recognized neurological risks associated with endovascular repair.

As a result of this acute neurological event, the patient was closely monitored and subsequently transferred to the neurosurgery ward after a two-week stay in the intensive care unit. Neurological examination revealed complete plegia of the right upper extremity, spontaneous movement in the left upper extremity, and symmetric but limited motor responses to painful stimuli in both lower extremities. After approximately one month of inpatient follow-up, the patient was discharged home in stable clinical condition.

## Discussion

The risk of iatrogenic aortic injury from spinal instrumentation is well documented; however, its management remains complex because of variability in the timing of diagnosis, presence of symptoms, and patient-specific risk factors.^2^ TEVAR is a rational approach for preventing catastrophic rupture during screw extraction in patients with instrumentation-related aortic involvement.^3, 4^ Although pre-removal TEVAR deployment is advocated in the literature as a means to ensure immediate control in case of rupture,^5^ our team opted for a staged approach after thorough multidisciplinary discussion. The graft was not deployed prior to screw removal to minimize the risk of endograft malposition or dislodgement during patient repositioning to the prone position. Furthermore, the exact depth of aortic penetration could not be confirmed radiologically, and deploying the stent without visual confirmation of screw mobility carried additional risk. To mitigate both scenarios, a TEVAR sheath was placed in advance for rapid deployment if rupture occurred during screw removal, which was performed under tightly controlled hemodynamic conditions. This approach was tailored to the patient’s specific anatomical and cardiovascular status. The presence of Takotsubo cardiomyopathy further complicates the perioperative course,^6^ as these patients are at high-risk for hemodynamic instability, arrhythmias, and low cardiac output, particularly during major vascular interventions. Anaesthetic management must balance pressor use to maintain coronary perfusion with strategies that prevent excessive afterload, which can exacerbate heart failure.

On postoperative day 2, the patient developed a right-sided middle cerebral artery infarction, which we attributed to a thromboembolic complication of the TEVAR procedure. Intraoperatively, 5000 IU of intravenous low molecular weight heparin was administered following stent graft placement as prophylaxis. Continuous ECG monitoring revealed no atrial fibrillation, and echocardiography excluded intracardiac thrombi. Given the infarct timing and distribution, distal embolization originating from the endovascular graft or its manipulation was considered the most likely source. Although rare, such neurological events following TEVAR are increasingly recognized and highlight the importance of optimized antithrombotic protocols and vigilant postoperative neurological assessment.^7^

## Conclusion

This case highlights the importance of vascular intervention in patients with instrumentation-related aortic involvement, particularly when a direct impingement is identified. A staged surgical approach with TEVAR before screw removal minimizes the risk of catastrophic hemorrhage. The presence of Takotsubo cardiomyopathy further complicates anaesthetic management, necessitating careful perioperative monitoring, goal-directed hemodynamic support, and multidisciplinary collaboration.

## Ethics

**Informed Consent:** Informed consent was obtained.
